# The therapeutic potential of gypenosides for age-related macular degeneration

**DOI:** 10.3389/fnut.2026.1773391

**Published:** 2026-02-04

**Authors:** Shuibin Cen, Jianping Li, James Reilly, Jinwei Chen, Hui-Rong Jiang, Xinhua Shu

**Affiliations:** 1 School of Materials, Guangdong Industry Polytechnic University, Guangzhou, Guangdong, China; 2 College of Basic Medicine, Guangdong Jiangmen Chinese Medicine College, Jiangmen, Guangdong, China; 3 Department of Biological and Biomedical Sciences, Glasgow Caledonian University, Glasgow, United Kingdom; 4 Strathclyde Institute of Pharmacy & Biomedical Sciences, University of Strathclyde, Glasgow, United Kingdom

**Keywords:** age-related macular degeneration, gut microbiome, gypenosides, inflammation, lipid metabolism, oxidative stress, therapeutic potential

## Introduction

**Figure 1:**
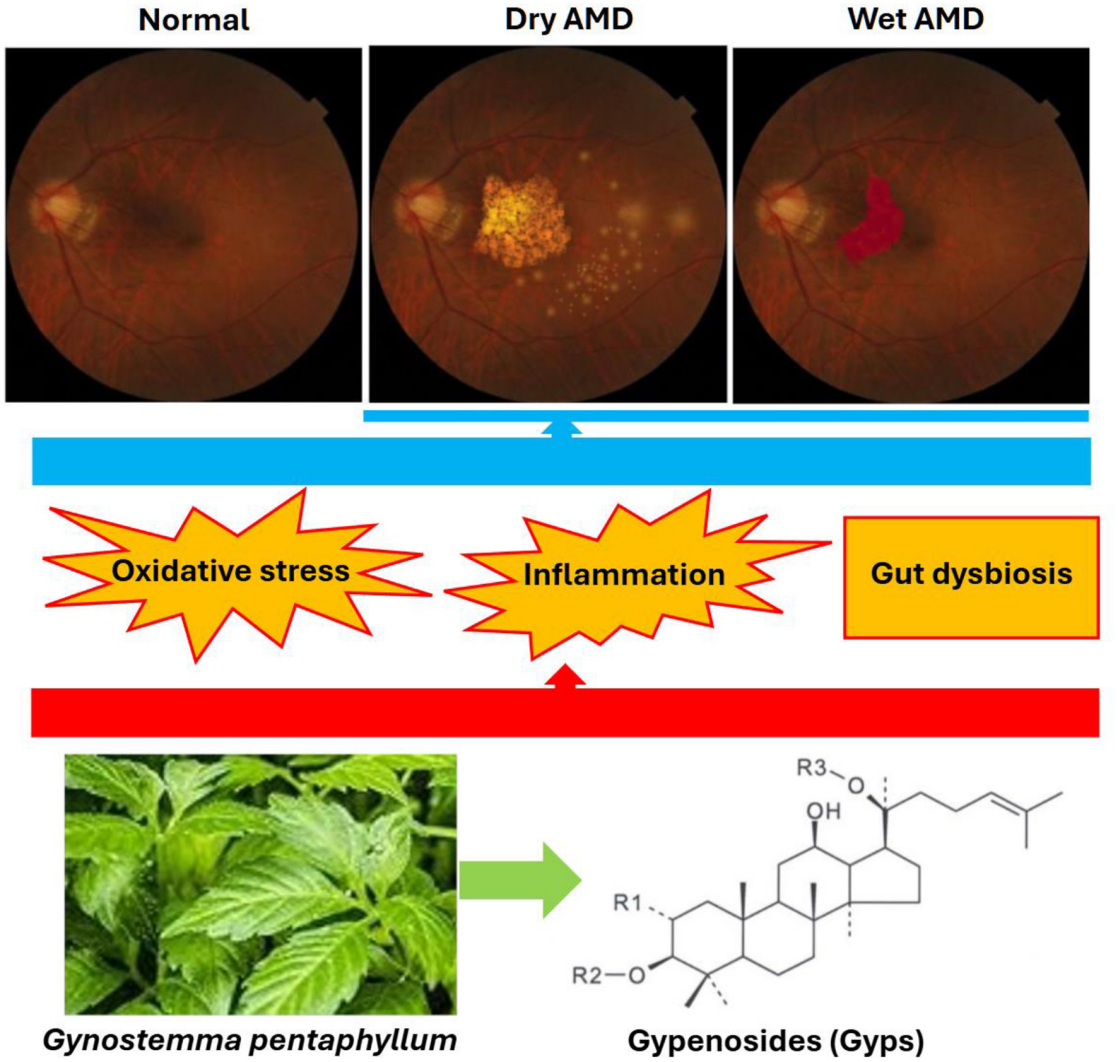
Age-related macular degeneration (AMD) is of two types: dry AMD (presence of geographic atrophy and drusen) and wet AMD (presence of choroidal neovascularization). Oxidative stress, inflammation, and gut dysbiosis are proposed to contribute to the pathogenesis and progression of AMD. Gypenosides (Gyps), a group of main active compounds in *Gynostemma pentaphyllum*, contain both the hydrophilic sugar part and the hydrophobic sapogenin part in the molecule (R1 and R2: glucose, rhamnose; R3: glucose, xylose). Gyps have been shown to suppress oxidative stress and inflammation and mitigate gut dysbiosis. Therefore, Gyps have the therapeutic potential for treating AMD.

Age-related macular degeneration (AMD) is a progressive retinal disease considered to be the leading cause of irreversible vision loss among older adults globally (1). AMD is classified into non-exudative (dry) AMD and neovascular (wet) AMD (Figure 1). Dry AMD is the commonest type, accounting for approximately 90% of cases and is characterized by atrophy of retinal pigment epithelial (RPE) cells and sub-RPE deposits (drusen); wet AMD is the most severe form and is characterized by the growth of abnormal choroidal vessels and leakage of blood and fluid (2, 3). Current treatments approved by the Food and Drug Administration (FDA) of the United States include anti-vascular endothelial growth factor (VEGF) treatment for wet AMD and anti-complement treatment for dry AMD, respectively (4, 5). These lifelong intravitreal treatments can cause adverse complications, including ocular hemorrhage, retinal inflammation, retinal detachment, and cataract (4, 5). Therefore, development of alternative treatments is urgently needed.

Gypenosides (Gyps), the triterpenoid saponins derived from *Gynostemma pentaphyllum* (*G. pentaphyllum*, called Jiaogulan in Chinese) (Figure 1), have demonstrated pharmacological activities, including inhibition of oxidative stress and inflammation and regulation of metabolism and immune response. In preclinical studies, Gyps have been shown to have protective effects against cancer, cardiovascular disease, diabetes, liver diseases, and neurodegenerative and neuropsychiatric disorders (6–8). In clinical trials, Gyps-rich extract from *G. pentaphyllum* has a beneficial effect against obesity, diabetes, anxiety and fatigue (9–12). Based on current findings on pathological mechanisms of AMD and pharmacological functions of Gyps, in this opinion piece we discuss the therapeutic potential of Gyps in treatment of AMD patients.

## Age-related macular degeneration (AMD)

### Overview of AMD

AMD primarily affects the macula, the central region of the retina, which is responsible for high acuity vision. The prevalence of AMD is estimated to be 8.69% in the global population between 45–85 years of age, affecting approximately 200 million individuals in 2020, rising to more than 288 million people by 2040, due to population aging (1). AMD is classified into three distinct stages according to the clinical progression. Early AMD is defined by the presence of medium-sized drusen (63–125 μm) only, intermediate AMD by the presence of medium-size drusen and pigmentary abnormalities or by large drusen (>127 μm) with/without pigmentary abnormalities, and late AMD by the presence of geographic atrophy and/or choroidal neovascularization (13). AMD pathogenesis and progression is associated with both environmental and genetic risk factors. Age is the major non-modifiable environmental risk factor, while other risk factors include smoking, high-fat diet (HFD), elevated body mass index, high levels of serum cholesterol, hypertension, and cardiovascular disease (14). Two major susceptible genes contributing to AMD are *Complement Factor H* (*CFH*) and *LOC387715*/*HTRA1*. Genes involved in lipid metabolism, collagen synthesis, extracellular matrix organization, and receptor-mediated endocytosis are also associated with AMD (15).

### Oxidative stress and inflammation are associated with AMD

It is proposed that the RPE plays a critical role in the pathogenesis of AMD. RPE cells have high metabolic activity and consume large amounts of oxygen, resulting in production of high levels of reactive oxygen species (ROS) (3, 16). ROS is also produced in RPE cells due to their role in the daily renewal of photoreceptor outer segments, as a result of which H_2_O_2_ is generated from β-oxidation of lipids in the shed outer segments and from the nicotinamide adenine dinucleotide phosphate (NADPH) oxidase in the phagosome (16). Additionally, light processing for vision also produces photooxidative stress, as earlier studies have shown that sun exposure is a risk factor for AMD (3, 16). Excessive ROS causes RPE dysfunction or even death by damage to intracellular lipids, proteins and mitochondrial DNA. Further, excessive ROS can activate the mitogen-activated protein kinase (MAPK) and nuclear factor kappa-light-chain-enhancer of activated B cells (NF-κB) pathways, resulting in production of proinflammatory cytokines, which exacerbate damage to RPE cells. Furthermore, excessive ROS or dyshomeostasis of antioxidant defense systems could stimulate VEGF production, resulting in formation of choroidal neovascularization, the hallmark of wet AMD (3, 16). Given RPE cells maintain photoreceptor function by supplying nutrition, secreting growth factors, phagocytosing the outer segments, recycling vitamin A, and forming the outer blood-retinal barrier, dysfunction/loss of RPE cells leads to progressive degeneration of photoreceptors.

### Nutrition and gut microbiome are associated with AMD

Nutrition plays an important role in AMD management, as specific nutrients (e.g., carotenoids) and dietary patterns (e.g., the Mediterranean diet, enriched with ω-3 fatty acids) have been shown to reduce the risk of AMD development and slow its progression (2). Nutrition is one of the crucial regulators of gut microbiota and directly influences gut microbial homeostasis and host health. Dysbiosis of gut microbiota is associated with development of AMD (17). Zinkernagel et al. (18) reported that the relative abundance of genera *Anaerotruncus* and *Oscillibacter* together with species *Ruminococcus torques* and *Eubacterium ventriosum* was significantly increased in gut microbiota of Swiss AMD patients, whereas species *Bacteroides eggerthii* was enriched in heathy individuals. Another study demonstrated that genera *Veillonella* and *Lactobacillus* were enriched, while genera *Faecalibacterium, Blautia, Anaerostipes, Anaerobutyricum*, Massilistercora, *Eggerthella, Megamonas*, and Desulfovibrio were depleted, in Chinese AMD patients, compared to healthy controls (19). The authors also reported 14 species were positively associated with AMD pathological features (fovea thickness and lesion size). Additionally, activities of 24 metabolic pathways were also positively associated with lesion size in AMD patients (19). Further, Parekh et al. (20) reported that composition of gut microbiome and gut-derived metabolites were different among the heath controls, intermediate and advanced AMD patients; taxa associated with immunologic functions were enriched in advanced AMD, compared to that of intermediate AMD, and the levels of some protective short chain fatty acids and bile acids were significantly lowered in the advanced AMD cohort, compared to the intermediate group. In AMD animal models, HFD or high-glycemia diet induced altered composition, diversity, and metabolic functional pathways of gut microbiome and exacerbated AMD-like pathologies (21–23).

## Pharmacological activities of gypenosides (Gyps)

### Gyps inhibit oxidative stress and inflammation

Gyps are a group of triterpenoid saponins, enriched in *G. pentaphyllum* which is widely distributed in some provinces of China as well as other South and East Asia countries. Around 250 Gyps have been identified, demonstrating a wide range of functions (6). Many previous studies have shown the capacity of Gyps against oxidative stress and inflammation among *in vitro* cell lines and *in vivo* animal models. For example, Gyps inhibited production of ROS and malondialdehyde (MDA) and increased activities of antioxidant enzymes in H_2_O_2_ or oxidized low-density lipoprotein (ox-LDL) exposed cell lines. Similarly, Gyps also decreased production of proinflammatory cytokines in ox-LDL or lipopolysaccharide (LPS) treated cell lines. The antioxidative and anti-inflammation function of Gyps was further confirmed in animal models of disease, such as atherosclerosis, ischemia-reperfusion injury and Alzheimer's disease rodent models. It is proposed that NRF2 and NF-kB pathways are involved in these functions (6, 24).

### Gyps regulate lipid metabolism

Gyps have also been demonstrated to regulate triglyceride and cholesterol metabolism (24). Gyp exposure can reduce cellular total triglycerides and cholesterol and increase lipid oxidation in cell lines (6). Treatment with Gyps reduced liver lipogenesis and enhanced lipid oxidation, resulting in a decrease in total blood triglycerides, cholesterol and LDL, and in liver lipid accumulation in HFD-fed mice (6). Clinical trials also showed Gyps' hypolipidemic effects. Individuals treated with Gyps had a significant decrease in total body weight, total fat mass, body mass index, and blood triglyceride levels. It is believed that Gyps regulate triglyceride homeostasis mainly via the PPAR/UCP1/PGC-1α/PRDM16 pathway (predominantly in fatty acid catabolism) and the SREBP-1c-ACC/FASN-CPT1 pathway (predominantly in triglyceride accumulation). Gyps regulate cholesterol homeostasis mainly via the SREBP2-HMGCR pathway (cholesterol biosynthesis), the PCSK9-LDLR pathway (degradation of LDL cholesterol), the LXRα/ABCA1/ABCG1 pathway (cholesterol transport), and the bile acid metabolism pathway (cholesterol excretion) (8).

### Gyps modulate gut microbiome

Previous studies also demonstrate that Gyps regulate gut microbiome and related metabolites in rodents. Gyp treatment has been shown to lower serum total triglycerides and cholesterol, decrease the ratio of *Firmicutes*/*Bacteroidetes* (a biomarker for metabolic disorders) and restore gut dysbiosis by increasing the abundance of health-beneficial bacteria and lowering the abundance of metabolic disorder-associated bacteria in HFD or high fat and high cholesterol (HFHC) fed mice (25–27). Similarly, Gyps was also shown to decrease total serum triglycerides and cholesterol, reduce the ratio of *Firmicutes*/*Bacteroidetes*, increase production of short-chain fatty acids, and alleviate bile acid metabolism in *ApoE* knockout mice fed with a high fat choline diet and in mice fed with HFD along with a single injection of streptozotocin (28, 29).

## Discussion

Our previous studies demonstrated that Gyps decreased the production of ROS and MDA, increased glutathione generation, and upregulated expression of antioxidant enzymes in H_2_O_2_-exposed human RPE cells. Gyps suppressed H_2_O_2_-induced production of proinflammatory cytokines at protein and mRNA levels in human RPE cells. Further experiments showed that Gyps' capacity against H_2_O_2_-induced oxidative stress, and inflammation was, respectively, through activation of the NRF2 signaling pathway and inactivation of the NF-kB pathways. Additionally, treatment with Gyps significantly reduced H_2_O_2_-induced apoptosis by inhibiting caspase activities (30). Our work in a zebrafish retinal disorder model also found that Gyps inhibited oxidative stress, endoplasmic reticulum stress and inflammation, and slowed down photoreceptor degeneration in disease zebrafish (31, 32). These data suggest that Gyps can restore RPE function under stress conditions and can promote photoreceptor survival, of significance in the treatment of AMD.

Gyps may also have therapeutic potential with respect to its effect on cholesterol metabolism. Dysregulation of cholesterol homeostasis is associated with a wide range of disorders, including cardiovascular diseases and Alzheimer's disease, and has been implicated in AMD (33). High-cholesterol diet increases the risk for the development of AMD (2) while animals fed with high-cholesterol diet display pathological characteristics of AMD (34). Abnormal accumulation of cholesterol and oxysterols has been detected beneath the RPE layer of AMD patients, suggesting dysregulation of cholesterol metabolism contributes to AMD pathogenesis (35). Genes related to cholesterol homeostasis, including *APOE, ABCA1, LIPC*, and *CETP*, are associated with AMD (15); knockout of certain cholesterol-related genes (e.g., *Abca1* and *ApoE*) in rodents causes retinal degeneration and recapitulates AMD pathological features (35). It is therefore reasonable to hypothesize that inhibition of cholesterol biosynthesis or enhancement of cholesterol metabolism or reverse cholesterol transport in RPE cells will benefit AMD patients. We have shown that Gyps upregulate expression of cholesterol trafficking and metabolism genes, promote cholesterol efflux and decrease lipogenesis of cholesterol, triglyceride and phospholipids in human RPE cells. Gyps also suppress uptake of ox-LDL and decrease intracellular ox-LDL level and ox-LDL-induced inflammation (36).

The therapeutic potential of Gyps against AMD needs to be further validated in AMD models. There are various mouse models that have been used to characterize the disease mechanism and evaluate the protective effects of drug candidates for AMD (37). To validate the therapeutic potential of Gyps, we would suggest choosing two mouse models: high fat high glucose (HFHG) diet induced model and *Sod*1^−/−^ model, as both models demonstrate key pathological features of AMD. Guided by previous publications (21, 23), study groups including the control, Gyp-treated, and untreated animals can be assessed for electroretinogram, retinal pathologies, oxidative stress, inflammation, abnormal accumulation of cholesterol and triglyceride, and lesion size of laser-induced choroidal neovascularization. Besides, neovascularization, and photoreceptor cell death in the retinal samples can be examined using biochemical and immunohistochemical approaches. Additionally, retinal metabolic and transcriptomic changes can be investigated by, respectively, liquid chromatography mass spectrometry (LC-MS) and RNA sequencing. Furthermore, the alteration of gut microbiome can be analyzed by 16 rRNA gene sequencing; changes in gut bacterial metabolites can be identified by LC-MS. Data from these experiments can provide further evidence of Gyps' therapeutic potential for AMD.

## Conclusion

This opinion article described the progress in the pathogenesis and treatment of AMD, summarized the pharmacological activities of Gyps, and discussed the therapeutic potential of Gyps for AMD (Figure 1). Preclinical studies in AMD animal models and clinical trials are warranted for confirmation of Gyps' beneficial effects against AMD.
